# A Review of the Field on Children’s Exposure to Environmental Contaminants: A Risk Assessment Approach

**DOI:** 10.3390/ijerph14030265

**Published:** 2017-03-04

**Authors:** Alesia Ferguson, Rosalind Penney, Helena Solo-Gabriele

**Affiliations:** 1 Department of Environmental and Occupational Health, Fay W. Boozman College of Public Health, University of Arkansas for Medical Sciences, 4301 West Markham, Slot 820, Little Rock, AR 72205, USA; AFerguson@uams.edu (A.F.); RBPenney@uams.edu (R.P.); 2 Department of Civil, Architectural, and Environmental Engineering, College of Engineering, University of Miami, Florida, 1251 Memorial Drive, Coral Gables, FL 33146, USA

**Keywords:** children’s exposures, life-stages, health, global burden of children’s diseases, environmental influences, children’s risk assessment

## Abstract

*Background*: Children must be recognized as a sensitive population based on having biological systems and organs in various stages of development. The processes of absorption, distribution, metabolism and elimination of environmental contaminants within a child’s body are considered less advanced than those of adults, making them more susceptible to disease outcomes following even small doses. Children’s unique activities of crawling and practicing increased hand-to-mouth ingestion also make them vulnerable to greater exposures by certain contaminants within specific environments. *Approach*: There is a need to review the field of children’s environmental exposures in order to understand trends and identify gaps in research, which may lead to better protection of this vulnerable and sensitive population. Therefore, explored here are previously published contemporary works in the broad area of children’s environmental exposures and potential impact on health from around the world. A discussion of children’s exposure to environmental contaminants is best organized under the last four steps of a risk assessment approach: hazard identification, dose-response assessment, exposure assessment (including children’s activity patterns) and risk characterization. We first consider the many exposure hazards that exist in the indoor and outdoor environments, and emerging contaminants of concern that may help guide the risk assessment process in identifying focus areas for children. A section on special diseases of concern is also included. *Conclusions*: The field of children’s exposures to environmental contaminants is broad. Although there are some well-studied areas offering much insight into children exposures, research is still needed to further our understanding of exposures to newer compounds, growing disease trends and the role of gene-environment interactions that modify adverse health outcomes. It is clear that behaviors of adults and children play a role in reducing or increasing a child’s exposure, where strategies to better communicate and implement risk modifying behaviors are needed, and can be more effective than implementing   changes in the physical environment.

## 1. Introduction

### 1.1. Studies of Children’s Exposure to Environmental Contaminants

The United States President’s Task Force on Environmental Health Risks and Safety Risks to Children has new 2016 endeavors to coordinate federal efforts to improve children’s health with particular focus on the areas of “asthma”, “chemical exposures and lead”, “climate change” and “the creation of healthy settings” [1]. However, 1996 marked the first monumental focus on children exposures to multiple chemicals following the United States Food Quality Protection Act in 1996 recognizing children’s vulnerabilities and sensitivities following aggregate and cumulative exposures to pesticides and other contaminants [2]. Since then there have been multiple efforts in multiple countries and continents to focus on reducing environmental exposures of children in order to improve health.

In any country, a number of agencies and institutions are chartered with the responsibility for children’s environmental health. In the United States, the Environmental Protection Agency (EPA) has the greatest duty for research and regulations concerning children’s exposures to environmental outdoor and indoor contaminants, while the Consumer Product Safety Commission (CPSC) oversees the safety of products marketed to children. Other agencies, such as the U.S. Food and Drug Administration (FDA), have a specific charter to ensure the safety of foods and drugs sold for consumption by children. Among these agencies, there can be an overlap in the regulation of products and chemicals used. Other non-governmental organizations such as the American Academy of Pediatrics, the American Lung Association, the Allergy and Asthma Foundation, and numerous university, state and local programs also have an impact on research and outreach to protect children from environmental exposures and adverse health outcomes. Those organizations affiliated with healthy lifestyles and healthy behaviors potentially play a role in reducing the impact of environmental exposures for children. The European Environment Agency is a dominant force in controlling environmental exposures for children in the member states of Europe; however, great variability is witnessed in other support systems and children’s health outcomes vary greatly across Europe [3]. Some well-known organizations in Europe that target environmental health for children include the European Commission, the European Public Health Alliance, and numerous European universities and smaller local organizations. Some developing countries cannot rely on such hosts of agencies, as other concerns at the forefront drain resources (e.g., civil unrest, extreme poverty). These areas rely greatly on the World Health Organization (WHO) and foreign aid (i.e., charity and health groups) to assist in children’s health and environmental initiatives [4].

A number of large studies have been established to examine the role of environmental exposures in children’s health. These include the Mother’s and Children’s Environmental Health (MOCEH) birth cohort study of 1220 subjects established in Korea since 2006 [5], the Maternal and Infant Research on Environmental Contaminants (MIREC) pregnancy cohort of 2000 subjects in Canada, and the Integrated Research Network in Perinatology of Quebec (IRNPQ) pregnancy cohort in Quebec of 5000 subjects [6]. In the United States, the National Children’s (NCS) Vanguard Study of 2009 followed 6000 children in 40 locations from pre-birth on to evaluate environmental influences on health [7]. The United States National Health and Nutrition Examination Survey (NHANES), in existence since 1960 provides valuable insights into exposures across multiple age groups, socio-economic categories, and geographic areas [8]. Although these large prospective birth cohort studies offer great insight into the potential role of environmental contaminants in children’s health, some challenges have been identified by Luo et al. These challenges include cost, quality control, limited data on early life exposures, heavy participant burdens, and lack of coordination and sharing of data across countries that could offer greater insight into population differences [6]. 

Although the environment has come to be understood as all-encompassing, this review focuses on unintentional and undesirable exposures to predominantly chemical agents, though on occasion we refer to some biological agents. Diseases linked to these environmental exposures are also explored. Fields of research on tobacco and alcohol use by children were not considered, nor were physical injuries. Healthy behaviors in terms of exercise and diet were considered in so far as they may affect the response to chemical and biological exposures in the environment. It is understood that undesirable exposures occur through the ingestion, inhalation, and dermal routes (i.e., aggregate exposures) for a child and that cumulative exposures (i.e., exposure to more than one chemical, sometime having the same toxicological endpoint) are possible and likely to occur. Aggregate and cumulative exposures are difficult to fully quantify, but must be considered for a comprehensive understanding of health outcomes. Within the risk assessment field cumulative exposures can also be understood as exposures to multiple adverse influences on health such as chemical agents, poverty and poor nutrition.

### 1.2. Children’s Exposures and the Risk Assessment Approach

As described by the U.S. EPA, the human risk assessment approach includes the five steps of planning and scoping, hazard identification, dose-response assessment, exposure assessment, and risk characterization. The risk assessment approach can also be represented in a more detailed format, and varying order as the health risk model that identifies the aspects of source, concentration, exposure, dose, and health outcome, with active corresponding links of fate and transport, activity patterns, uptake, and distribution, metabolism and elimination in the body [9,10]. Any improvements in individual steps of the human risk assessment approach improves the overall health risk estimate for children [11]. 

**Figure 1:**
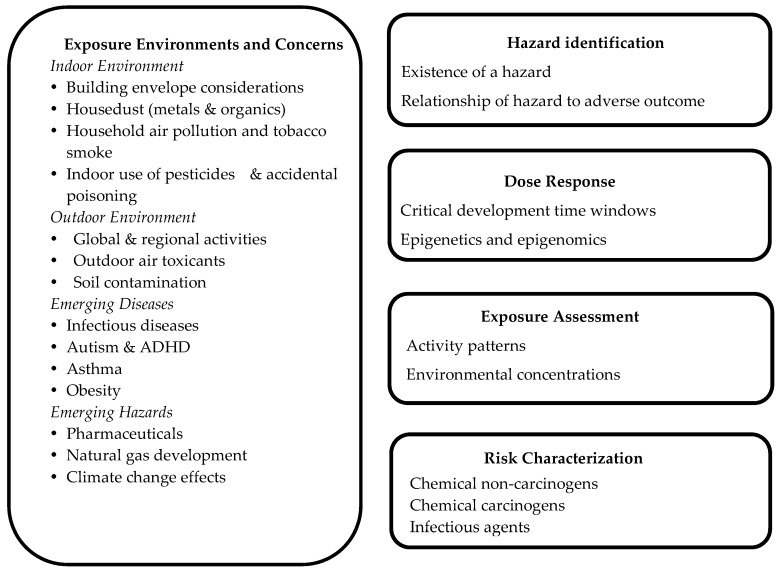
Key elements of risk assessment for children to evaluate exposure to environmental contaminants.

For consistency in the field, the difference between an exposure and a dose estimate must be clarified when discussing the following: evaluating health risks of environmental contaminants for children, distinguishing the dose-response assessment versus exposure assessment, and assessing the implication of study methods and outcomes. “Exposure” refers to the amount of a chemical agent that typically contacts the outer boundary of the body (i.e., human interface such as the skin), while “dose” refers to the amount of a chemical that crosses the human interface into the body and is a function of exposure [12,13]. A discussion of children’s exposure to environmental contaminants is best organized under the last four steps of a risk assessment approach: hazard identification, dose-response assessment, exposure assessment (including children’s activity patterns) and risk characterization. We first consider the many exposure hazards (i.e., potential exposure environments and agents) that many exist in the indoor and outdoor environments, and emerging contaminants of concern that may help guide the risk assessment process in identifying focus areas for children. A section on special diseases of concern is also included (Figure 1).

Planning and scoping, the first step of a human risk assessment, must not be disregarded in preparing a project and making decision on the scope, breadth, and approach in addressing an exposure or health concern for children. Successful planning and understanding of all components of the risk assessment strategy will lead to more applicable and successful study outcomes. Although all aspects of planning and scoping are not discussed in this review article, it is noted that many aspects of planning and scoping also rely of program goals and timelines, cost considerations and personnel capabilities, within an organization planning a children’s study [14].

## 2. Approach

### 2.1. Exposure Environments and Concerns

Here we look at exposures that can occur in the indoor environment, outdoor environment, along with environmental exposures as a result of emerging contaminants and chemical and biological hazards posed by climate change. We also take a look at special diseases of interest for children and the factors that may play a role.

#### 2.1.1. Hazards in the Indoor Environment

The building envelope can protect a child’s health by minimizing constant exposure to outdoor pollutants and extremes in temperatures. However, the availability and the concept of housing and home differ greatly among developed and developing countries, and therefore time spent in homes and the exposures experienced will vary. The structure of the building envelope is a driver for outdoor infiltration, however the variety and use of indoor products will heavily determine the contaminant load indoors [15,16]. Regrettably, there is less control and understanding of the residential home environment due to these great variations, and there is restricted access for researchers and officials. Regulators in some countries attempt to control products brought into the home and some activities of contractors through building, plumbing, electrical, heating and air, and renovation codes [17]. In the United States and Europe, common in-home exposures of concern for children include lead, radon, pesticides, and tobacco smoke; growing concern exists for mold, volatiles from consumer and building products (e.g., formaldehyde, naphthalene, benzene, ammonia), and household allergens (e.g., house dust mites, cockroaches, rodents) [18,19,20]. The growing concerns of in-home exposures exist not only because environments are changing (e.g., new and growing product usage, more closed environments), but because of new research findings on adverse health outcomes even at low exposure levels. Concerns for energy efficiency in some areas of the world have led to a tightening of the building envelope, which can lead to the increased trapping of volatile pollutants indoors when proper ventilation is not provided [21]. Children’s play activities and household cleaning activities will also greatly influence exposures for the child in the home. Interventionby educating families and individuals to alter behavior is also important to lessening environmental exposures in the home and improving health [22]. 

Children’s exposure to housedust is of particular concern due to the various contaminants found in housedust and children’s activity patterns of crawling on floor surfaces and practicing pica-activity that increase opportunities for exposure and uptake. A host of metals and organics are found in housedust where sources include track-in of outdoor soils, gas and aerosol infiltration, release from products brought into the home, new building and old building materials [23,24,25]. Although, there have been increased regulations to protect children from lead exposure in most countries, various sources of lead continue to contribute to children’s adverse health outcomes following lead poisoning. Here in the United States lead-based paint found in homes built before 1978 still leads to contaminated lead dust in the home [26]. With the increased knowledge of adverse health outcomes at low levels of exposure, the blood lead awareness level for children was lowered to 5 µg/dL in 2012 in the United States and it is estimated that 2.5% of children are at this level and above following the 2007 to 2010 NHANES data [27]. Researchers continue to look at the nuances of learning deficiencies and other adverse outcomes for children following lead poisoning and the influence for minority or low socio-economic groups that might be more heavily impacted [28]. Some countries can still see higher exposures from industries where little regulation exists and where leaded gasoline is still used, resulting in more expansive environmental contamination and poisoning outcomes for children [29].

More extreme household air pollution (HAP) effects continue to plague areas that lack electricity, given that over 2.8 billion people globally rely on solid fuels for cooking [30]. The WHO estimates that over 4 million premature deaths result from the indoor use of solid fuel, where children and those who are compromised suffer the most [31]. A review revealed studies with associations between HAP and acute lower respiratory infections, fatal and non-fatal pneumonia, stunting, adverse pregnancy outcomes, and other-cause mortalities [32]. The studies also demonstrated that sustained interventions (e.g., cleaner fuels, better stoves, increased ventilation) are challenging in these environments. Often, these environmental exposures and adverse outcomes are made worse by poverty, poor nutrition [4,33] and the presence of other childhood communicable and non-communicable diseases [34]. HAP resembles the toxicity of traffic and industrial pollutants, dependent on the type of fuel used for cooking or for lighting use in the home (e.g., kerosene lamps) [35]. Exposures to polycyclic aromatic hydrocarbons (PAHs), one class of compounds generated from incomplete combustion resulting from activities or events such as domestic wood burning, power plants, and forest fires, continue to concern us for both the indoor and outdoor environment globally. PAH exposures in household dust during children’s critical windows have been linked to a number of adverse outcomes in children, including childhood acute lymphoblastic leukemia [36]. 

The adverse respiratory and carcinogenic effects of second-hand and, increasingly, third-hand smoke continues to concern public health officials as an avoidable environmental exposure indoors for children (both pre- and postnatal exposures) that lead to lung cancer, motor impairment, hearing and vision problems, cardiovascular consequences, and learning disabilities [37,38,39]. Disparities in exposures continue to exist, where children from minority backgrounds and those in low socio-economic groups experience higher exposures to second-hand tobacco smoke not only in the United but in other countries [39,40]. Children’s exposures to tobacco smoke and other contaminants that occur in multi-unit apartment buildings also pose particular challenges to control outside of established smoke-free policies in the United States [41]. The smoking rates, smoking cultures and strength in smoking policies vary across countries and even localities, therefore continued interventions are needed to protect children, including parental education, taxations, smart strategies for smoke-free policies for the home, school-based educational programs, and improved indoor ventilation [42]. Although electronic cigarettes may be a healthier alternative to cigarettes in terms of absence of carcinogenic combustion by-products, many researchers are still concerned with its use as a gateway drug, and adverse health outcome in terms of propylene glycol content, other chemical content, voltage risks, accidental poisoning from cartridges, and any existing side effects of nicotine [43]. More research on electronic cigarettes is encouraged where children are concerned. 

Pesticides encompass a wide class of compounds (e.g., insecticides, herbicides and fungicides) used to eliminate unwanted pests (including plants) across farms, homes and public settings where children spend time (e.g., schools and parks). Changes in the types of pesticides and recommendation for their usage has also occurred as researchers have discovered the potential adverse outcomes for reproductive, neurological and carcinogenic outcomes, especially for children and pregnant women [44,45,46,47]. Efforts have been focused on finding safer pesticides for the home (e.g., the replacement of organophosphates and organochlorines with plant-based or synthetic pyrethroids), although even these newer compounds are under scrutiny for suspected allergic and asthmatic outcomes [19,48,49]. Efforts have also been focused on reducing pesticide residue track-in from harsher pesticides used agriculturally, and providing other protective measures for Latino farmworkers and their children [50]. 

Accidental poisonings in children can be viewed as environmental exposures and are more dominant in the home and between the ages of 1–5 years [51]. In a study of 489 children aged 1–17 admitted to the emergency room in Ankara between 1995 and 2000, Andiran and Sarikayal found accidental poisoning in 97.1% of the 331 children under 5 years of age, where drugs were the most common agent, followed by corrosive substances, and carbon monoxide poisoning [51]. Acute pesticide poisonings are also concerning in many countries [4]. In Ecuador, Costa Rica, and Nicaragua, organophosphate and carbamate pesticides are the common agents for acute poisoning in children [4]. This demonstrates the importance of recognizing exposures in the home and implementing safety steps to protect children (e.g., proper storage and disposal of chemical agents, and the maintenance of gas burning appliances). 

#### 2.1.2. Hazards in the Outdoor Environment

Outdoor exposures for children vary greatly and can be affected by regional and global activities, along with climate and topographical considerations that lead to air, water and soil contamination by heavy metals, pesticides, various classes of volatiles, and biological contaminants. Regional and global activities that influence outdoor exposures include natural disasters (e.g., earthquakes and hurricanes) and manmade influences (e.g., traffic, improper waste disposal, agriculture, mining, and smelting) [4]. In the United States, air pollution has decreased in the last 40 years following increased restrictions for car emissions, coal and gas refineries, and other manufacturing industries. Despite this, some urban outdoor pollution levels are still considered dangerous to breathe [52]. With population increase and industrialization in developing countries, global air pollution is on the rise [4,15]. Some developing countries, such as the Latin American countries, report greater environmental and air pollution effects from climate change and deforestation activities [4]. Based on its Global Health Observatory, the WHO estimates that 3.7 million premature deaths in 2012 were attributable to ambient air pollution, where 88% of that burden occurred in low and middle income countries [31]. In particular, WHO finds that 3% of these premature deaths in children are due to acute lower-respiratory infections.

Outdoor air toxicants of concern for children’s respiratory and general health (e.g., asthma, COPD, respiratory and other cancers, and neurological and reproductive damage) around the world continue to be the criteria pollutants as defined and monitored by U.S. EPA (i.e., ozone, particulate matter, carbon monoxide, sulfur dioxide, nitrogen dioxide, and lead) [15,53]. Growing pollution from other heavy metals, dioxins, benzene, pyrene, and PAHs are also of concern and vary globally and are influenced by consumer product production and heating needs, and proximity to traffic [54]. Including decrease in lung function, air pollution further plays a role in the increase in ear and respiratory infections for children [55] where the critical window of exposure plays a role in the significance of the association [15]. Ambient air pollution has also recently been linked to male gamete damage in a number of studies where further research is needed on the relevant exposure conditions, contributing factors, and clinical mechanisms for these newer health outcomes [56]. Young children spend significantly more time than adults in outdoor play areas, especially in warmer climates and particular cultures. Although inhalation exposure is the primary concern in outdoor environments, soils in playgrounds, parks and beaches may be contaminated with air pollutants like the metals arsenic and lead [57,58], putting children at risk for exposure also through the dermal and non-dietary ingestion exposure routes. 

### 2.2. Some Special Diseases of Concern in the 21st Century

Highlighted here are only a number of common or growing diseases of concern globally for children. The identification of trends and severity of diseases falls into the efforts of hazard identification for the human health risk assessment model. 

#### 2.2.1. Infectious Diseases

Bacterial, viral, and other infectious exposures are an immediate concern for children in some regions where water quality and sanitation infrastructure are poor [59]. In 2010, there were 7.77 million deaths globally due to diarrhea, lower respiratory infections, meningitis, and other common infectious diseases, and 0.211 million deaths for neglected tropical diseases and malaria [60]. Sexually transmitted diseases and hepatitis are not included in that count. Foodborne bacterial, protozoal, and viral diseases alone are estimated to result in two billion cases worldwide and one million deaths, where children bear the considerable burden of infectious diseases [61]. Even developed countries are challenged by some viral and bacterial infections; in the United States, for example, the leading foodborne disease-norovirus causes 570–800 deaths a year, where food workers in restaurants are the group believed to be primarily responsible for disease transmission [62]. Norovirus alone was responsible for 1 million medical care visits for children under 5 years of age from 2009 to 2010 in the United States [63].

Due to ecological and infrastructure damage, children in communities affected by natural disasters are more prone to increased exposures to a host of environmental contaminants (e.g., cholera outbreak following the Haiti earthquake of 2010) [64]. The concern for viral and bacterial transmission agents can also be unique to some localities and regions. For example, dengue and malaria have been on the rise over the last six years, likely due to increases in mosquito breeding sites [65]. The Zika virus, also transmitted by mosquitos, has become a concern for its link to microcephaly and other birth defects [66]. Environmental controls focused on eliminating mosquito breeding sites and reducing transmission routes will be crucial as Zika cases has increased in Brazil and has also spread to over 47 countries and territories, including most recently to the U.S., within the subtropical regions of Miami, Florida [66,67]. Additionally, children’s play areas specifically can be a cause for concern for infectious disease transmission. Human bacterial pathogens are present in water fountains of parks from where children drink [68] and infectious disease agents are found in sands where children play, such as at beaches and parks [69,70,71]. 

#### 2.2.2. Asthma and Allergies

The rates of asthma and allergies continue to grow [72,73,74], where a combination of factors have played a role in the increasing rates, including increased rates of diagnosis [75]. For asthma, there are some common findings among studies for causal and related factors, such as positive association of a child’s asthma outcome with atopy, parental atopy and asthma, indoor and outdoor pollution, and a negative association with living in a rural area [53,73,74,76,77,78]. The Institute of Medicine, based on a wealth of studies, found that respiratory syncytial virus (RSV), house dust mite (HDM) allergens, cockroach allergens, and cigarette smoke may play a strong role in the development of asthma, while these and many other biological, and chemical agents act as triggers [79,80]. Successful intervention studies that target triggers and causal factors for asthma and allergies inside the home and in school environments have been reported [81]. Earlier work from Halken’s group has shown great benefits in the reduction of allergic disease in children by controlling both the environment—including exposure cigarette smoke, HDM, and pets-and infant diet in the first 4 to 6 months [82]. 

Gene-environment interactions also continue to be explored by researchers for the development and aggravation of asthma and allergies. For example, variants at chromosome 17q21 show strong associations on exposure to maternal smoking [83], however, the interaction between air pollution and genes for incident asthma show mixed results in a number of studies [76]. Likewise, the manifestation of atopic dermatitis (AD) in children appears to be on the rise in many countries, and the potential environmental triggers may be dependent on ethnic (race, culture and place of birth) differences, where typically Blacks and non-Hispanic whites show higher AD rates [84,85]. Filaggrin, a filament-associated protein, has been identified most recently as associated with a host of allergic diseases, including AD and asthma [85]. Important opportunities exist to expand related work and discover cause-and-effect relationships along with multifaceted interventions. Some researchers, however, propose that since some legacy environmental contaminant concentrations are not on the rise, other factors are at play; changes in theenvironment might be attributable to the rising rates of asthma and allergies, such as changes in diet, cleaner environments for infants and children (i.e., “hygiene hypothesis”), growing obesity rates, and increased use of some medications that result in immune deficiencies or damage [86]. Leonard et al. recently published work exploring how the gut microbiome may play a role in a host of autoimmune diseases, including celiac disease [87]. 

#### 2.2.3. Attention Deficit Hyperactivity Disorder and Autism

Across multiple studies, various positive and negative findings demonstrate links between Attention Deficit Hyperactivity Disorder (ADHD) and environmental pre- and postnatal exposures (i.e., metals, pesticides, tobacco smoke, PCBs) [88,89]. Based on data from the 1999–2002 NHANES study for 4,704 children aged 4–15, prenatal tobacco smoke and lead poisoning were significantly associated with ADHD [90]. However, genetics are believed to be involved in 75% of ADHD cases, dictating both the level of severity and persistence of the condition into adulthood [91]. 

Autism is another neurological disease for which environmental exposures such as PCBs, PBDEs, heavy metals, pesticides, and biological agents are believed to play a role, and for which comorbidities, such as immune system dysfunction and other psychiatric problems, are common; it is believed autism has been on the rise over the last 20 years in the United States and around the world [75,92]. In a 2014 review, Lyall et al. focused on modifiable factors such as nutrients and supplements taking by the mother (e.g., folic acid, especially in the presence of certain gene variants), along with smoking and alcohol use, concluding that these factors require further exploration [93]. Again, future studies examining gene-environment interactions, multiple chemical and biological exposures, critical windows for exposure for the mother and child, and child psychosocial interactions could provide improved casual understandings of ADHD, autism, and other neurological childhood diseases [75,88,93].

#### 2.2.4. Childhood Obesity

Obesity rates continue to grow in developed and developing countries [65]. A recent article from the UK Millennium Cohort Study related obesity to the early life inequality factors of maternal smoking during pregnancy, maternal pre-pregnancy overweightness, and low educational attainment as predictors [94]. These predictors may provide some understanding for the varying rates of obesity in certain socio-economic populations, however it is well understood that changes in dietary intake and levels of physical activity related to caloric imbalances are the leading causes for obesity, especially in the industrialized world [95]. In recent epidemiological studies, obesity has also been attributed to endocrine disruption [96]. Endocrine disrupting chemicals, including prenatal exposures to bisphenol A, phthalate metabolites, and organochlorine pesticides, are suspected of causing changes in cell signaling, which ultimately affect adipocyte differentiation and energy storage. However, animal and epidemiological studies in this field, along with studies that more broadly address the potential epigenetic and epigenomic changes that may be involved in the obesity epidemic, are new and inconsistent in their findings [5,96,97,98,99,100]. Indeed, compounds like bisphenol A and phthalates are concerning and they appear ubiquitous in various environments (i.e., indoors and outdoors) and in human populations in the United States [98] and all over the world [5]. 

Obesity itself has now been associated with a variety of autoimmune and inflammatory diseases in the industrialized world, including multiple sclerosis, inflammatory bowel disease, and rheumatoid arthritis [101]. The host of potential impacts of obesity on a child’s health is complex. The relationship to diabetes is well-established [102], but questions arise as to whether obesity can lead to an altered response to exposure to even lower concentrations of environmental chemicals by affecting processes of biological distribution, metabolism, and elimination, as is seen in drug pharmacokinetics [101,103]. Because obesity is linked to poor diets, lower intake of protective antioxidants may also be occurring. The obese and overweight tend to participate less in physical activity outdoors, leading to more sedentary lifestyles in the home environment and potentially greater exposures where the characteristics of indoor pollutants are changing. 

### 2.3. Emerging Hazards

Below is an introductory discussion of emerging environmental exposure concerns. Here we address some emerging contaminants and the adverse and expected influence of climate change on the environment and potentially on exposures for children.

#### 2.3.1. Emerging Contaminants

Our water and food sources are increasingly becoming contaminated with other emerging contaminants, including pharmaceutical and other new manmade compounds [104]. A recent study found concentrations of pharmaceuticals and other organic waste compounds in 44 domestic drinking water wells in Cape Cod Massachusetts [105]. Acesulfame is a potential marker for such organic waste compounds (OWC), given its persistence and common use in products such as foods, beverages, and toothpaste [105]. A recent study in Israel showed concerning levels of uptake of some pharmaceutical compounds by root vegetables (e.g., carrots) treated with wastewater [106], while other studies have provided little evidence of significant contaminant transfer from soil to various plants [107], demonstrating the need for further research into potential health links. Overloading of the environment by bisphenol-A and similar compounds used in plastics, is also concerning [99]. 

The United States has seen a rapid increase in unconventional natural gas development, predominantly around the Marcellus Shale in Pennsylvania. Growing concerns surround the impact of exposures to the pollutants generated in this process. Based on distance of homes to natural gas injection mines and the timing of various related mining activities, researchers found an association with preterm birth in a retrospective cohort study of 9,384 mothers over a 4-year period [108]. No association was found with other measures, such as gestational age at birth or term birth weight. It is expected that the field of children’s health will see many more related studies of this kind, moving beyond the environmental impacts of natural gas development to the study of human and, in particular, children’s health effects. Determining pathways of exposures (e.g., breast milk), drug and chemical interactions, concentrations of concern for sensitive populations like children, and strategies for pollution prevention and treatment will be areas for future research for all emerging contaminants [104].

#### 2.3.2. Climate Change Effects

Extreme climates impact human health in numerous ways, where climate change effects will bring a host of growing environmental and public health concerns. Increases in ambient temperatures, caused by climate change for example may increase preterm birth rates during critical windows of pregnancy, potentially more relevant for developing countries without air conditioned homes [109,110]. Longer periods of heat, brought on by climate change, will also promote bacterial and viral growth through vector growth (e.g., mosquitos, ticks), and will impact children’s health now and in the future [21,111,112]. Climate change effects, made worse by deforestation activities in some countries, leads to land degradation, contamination, and flooding therefore further promoting vector and microbial growth [4]. The United States has enjoyed improvements in air quality since the 1970’s largely with the implementation of the Clean Air Act and stricter emission regulations for the energy, industrial and transportation sectors. Despite this, climate change effects such as changes in ambient temperatures and extreme weather conditions can drive up energy usage and lead to increased fuel consumption and air emissions [113]. In addition, extreme drought conditions can lead to arid conditions and increased air pollution through wildfires, dry wind storms, and changes in types of aeroallergens (i.e., plant and animal species changes), all leading to increased adverse respiratory conditions for the very young and compromised across the globe [113,114]. The children of Southern Africa are uniquely susceptible to climate change effects, where this semi-arid region is becoming increasingly affected by droughts, flood, crop destabilization, and disease outbreak (e.g., cholera) [115]. 

The 2016 U.S. Global Climate Change Program identified a number of populations (e.g., minority and indigenous groups, children and pregnant women) with increased vulnerability to climate change impacts [116]. For children, temperature related deaths and illnesses will likely rise, where for example asthmatic children are affected by the increased frequency of wildfires and resulting particulate exposures. In addition, children are at greater risks for distress and anxiety caused by heat waves and other extreme events that interrupt normal outdoor play or result in life changing events (e.g., destruction of home and migration). We will likely see an emergence of substantial research in the area of climate change effects on air and water pollution and other environmental and public health outcomes for children [117]. 

## 3. Results and Discussion

### 3.1. Hazard Identification for Children

For hazard identification, expert judgment is used to determine existence of a hazard and the association to an adverse outcome based on some form of reasonable evidence [118]. Hazard identification can also involve a preemptive approach (i.e., avoiding known or trying to understand potential hazards) or can be in response to an adverse health outcome in the population (i.e., retrospective look at causal factors [i.e., hazards] and sources and pathways of exposure). Hazard identification justifies the reason to continue investigations along the risk assessment paradigm. In terms of a preemptive approach, sometimes there is a necessity to prioritize the chemicals that provide the greatest risks to children given limited resources and the need to more rapidly advance the protection of children’s health. Armstrong et al. suggested a formalized tiered approach that required addressing whether that chemical was found in large enough quantities in children’s environments, the chemical’s level of toxicity, and the potential activity patterns and exposure scenarios for children [119]. Georgopoulos et al. used a slightly different approach in ranking chemical exposures based on key environmental, demographic and socio-economic data [120]. Researchers have looked at more specific exposure scenarios, but with some prior understanding of the particular hazards. Driver et al. presented algorithms for children’s non-dietary exposures to pesticides applied indoors [121]; and Smith et al. used a scoring method to prioritize chemicals for toxicological analyses [122]. It is beneficial to have some general understanding of environmental hazards that exist in the environment and to look ahead at hazards that may present themselves in the future. The previous section on *environmental exposures and concerns* can provide researchers with some guidance in that regard.

### 3.2. Dose-Response Assessment for Children

Researchers are interested in any adverse response of a child’s body/organ/tissue to a certain dose of an environmental contaminant. Dose-response curves to environmental contaminants are generated from human studies or extrapolated from in vivo or in vitro animal studies, where considerations are needed for the unique toxicological response for children of varying ages. It is however useful to describe childhood as a life stage through which everyone must pass, where protection of the young results in protection of all through subsequent life stages.

In 2010, Hines et al., published on the risk considerations for sensitive and vulnerable groups (e.g., pediatric populations), highlighting works in the literature from 1996 to 2007 that addressed needs and findings on critical biological, and toxicological exposure related factors, methodological approaches, models or experimental designs, unique parameters, key gaps, and lessons learned [123]. The EPA also provides a wealth of documents related to risk assessment. Researchers might be particularly interested in *Guidance on Selecting Age Groups for Monitoring and Assessing Childhood Exposures to Environmental Contaminants* [124], as well as the U.S. EPA *Supplemental Guidance for Assessing Susceptibility from Early-Life Exposure to Carcinogens* [125]. Physiologically Based, Pharmacokinetic (PBPK) models utilize exposure inputs with distribution, elimination, and metabolic parameters can help the researcher estimate chemical concentrations in organs and tissues allowing us to begin to address dose-response and potential toxicological endpoints [123], when combined with relevant clinical data and an understanding of biologically effective doses that lead to an adverse response. PBPK models, therefore offer us an alternative to in vivo or in vitro studies, however often they rely heavily on parameter estimations from various sources (i.e., in vivo and in-vitro studies) as initial inputs and reasonable assumptions (i.e., partitioning dynamics based on chemical and tissue properties). In addition, there are limited PBPK models that incorporate life-stage appropriate data for children’s health outcomes. 

For an effective discussion of dose-response assessment, the logical approach is a dialogue concerning children’s developmental stages and the influence of exposures on health outcome, during these life-stages. The United Nations Convention on the Rights of the Child defines a child as a human under 18 years, unless otherwise noted in a particular country’s laws [126]. Often the research before 2000 focused on of two aspects: (1) comparing exposures in children to those in adults or (2) comparing simply prenatal versus postnatal exposure timeframes. In the late 1990s, several research working groups were created and consequently tasked with identifying critical windows in child development. These windows identify susceptibility to certain toxic agents to which a child may not be susceptible in another window [127]. The working groups were focused in five areas: cardiovascular and endocrine systems, immune and respiratory systems, neurobehavioral, reproductive health, and cancer. These groups’ findings were published in 2000 [128,129,130,131,132]. Several groups were able to more easily identify critical windows. Reproductive windows, for example, demonstrate that exposures in the parents in the pre-conceptual window can affect the genetics of the child, and that exposures can also alter the reproductive function in the pre-puberty/puberty window [131]. Some of the working groups found that these windows can be quite elusive. Nevertheless, subsequent studies are considering these critical windows and improving our understanding of children’s sensitivities in order to improve and establish protocols for health risk assessments and toxicity testing for consumer products [123,133,134]. Windows within other identified windows are also suggested. For example, Goldizen et al. recently considered five critical stages for gestational lung development (i.e., embryonic, pseudo-glandular, canalicular, saccular, alveolar) important for air pollution and respiratory effects following exposure of the mother [15].

There are other aspects of exposure in children, from a dose-response perspective, that also needs to be examined; several articles have summarized many of these considerations for studying this population. The 2006 EPA publication *A Framework for Assessing Health Risks of Environmental Exposures to Children* describes knowledge of exposure, critical windows of development for organ systems, modes of action, anatomy, physiology, and behavior as aspects of consideration when exploring health outcomes in children [11]. While not listed in the EPA publication, gene and genetic effects have emerged as important considerations as well. Understandably, epigenetics and epigenomics have developed as fields that refer specifically to the environmental insult in a key window of susceptibility that may promote negative neuro-developmental outcomes and deregulate signaling pathways that can affect various adverse health outcomes [75,100]. Epigenetic modifications are defined as those affecting phenotypic outcomes while more generational epigenomic modifications affect the genotype [100]. These studies then consider differences in genetic makeup between groups and individuals, where any gene-environment interaction may influence the toxico-kinetics of environmental contaminants, outcome of research findings, and the conclusions that are drawn for children’s health. For example, Makenure et al. identified an interaction between lung function and nitric oxide and nitrogen dioxide in African children with polymorphisms in the CD14 gene, with the variant being protective to the exposure [135]. In another study, polymorphisms in genes on the chromosome 17q21 locus were associated with wheeze and asthma in children living in rural areas [136]. As another example, neuro-developmental damage due to heavy metal exposures may change based on gene variants, including maternal-fetal gene variation [137]. Chow et al. found that cancers (except for acute lymphoblastic leukemia) were 28% more common in black children than white in the United States based on 13,249 cancer cases and 36,996 controls from five U.S. states between 1978 and 2004 [138]. However, the ultimate role that environmental exposures, genes, diet, and social conditions play in the etiology and mechanistic development of various cancers, pre-term birth, and other childhood diseases may be difficult to determine [139]. 

Physiological differences (e.g., heart rate, blood flow, and metabolism) can also be linked to differing pharmacokinetics in different age groups. Physiological differences (e.g., heart rate, blood flow, and metabolism) can also be linked to differing pharmacokinetics and possible adverse drug reactions in different age groups, especially in the early months after birth. Renal clearance may be affected by a decreased blood flow in the first few months after birth, and immature metabolic systems in this same age group can decrease metabolic rates. These rates can then increase to those greater than an adult as the system matures [140]. Ginsberg et al. identified a large fluctuation in certain drug half-lives up to over 4 times that of adults in neonates, to shorter than that of an adult from 6 – 24 months, and to that equal an adult’s from 24 months on. An inverse pattern of clearance in these age groups is also demonstrated [141]. While these discoveries begin to identify critical age windows, there is still much to be explored in this area. The physiologic changes that occur as a child develops from the prenatal stage to adulthood can cause adverse health outcomes from smaller doses of harmful compounds, such as chlordane, than would affect an adult. Much like lead, chlordane is a developmental immunotoxicant that has *in utero* effects at doses that have no effect on the immune system of the mother [130]. Kutanzi et al., in a review of pediatric exposures to ionizing radiation highlighted the potential adverse health outcomes (e.g., cancers) for children following exposures to low concentration of accidental and technological radiation, implying care when treating with diagnostic radiation considering the sensitivities of children and the potential role of genetic differences [142].

### 3.3. Exposure Assessment for Children

Direct (e.g., air monitors, skin patches) and indirect (e.g., algorithms, models) approaches are used by researchers to determine exposure and sometimes the combined estimate of exposure and dose. A direct measurement approach offers a look at exposure typically over a shorter period of time and may have some limitations in using children as study subjects (e.g., monitoring device cumbersome, unnecessary exposure to toxic study compound). A modeling approach uses information on activity patterns, environmental concentrations and other exposure parameters (e.g., measures or estimated adherence and deposition factors), and offers flexibility in addressing long term exposures and looking at parameter sensitivities [9,143]. Authors Young et al. have compared a number of probabilistic models (e.g., SHEDS, CARES) that were used to evaluate aggregate exposure to pesticides, and these can be adapted to address exposure to other environmental agents [144]. Regardless of assessment approach, exposure is largely affected by a child’s behavior (i.e., activity patterns) and concentration of the agent of concern in the environment as discussed below. In this section, biomarkers of exposure are also discussed.

#### 3.3.1. Determining Children’s Activity Patterns

Challenges exist in accurately determining children’s activity patterns for use in determining their aggregate (i.e., ingestion, inhalation and dermal exposures) and cumulative (i.e., exposures to more than one compound having the same toxicological endpoint). For one, the activities of children change as they age. Firestone et al. illustrated the role of age related behavior (such as mouthing, sleeping, and eating) on childhood exposure [145], while Holsapple et al. identified key physiological (heart rate, breathing rate, etc.) and physical (water and juice intake, fruit consumption, meat consumption, etc.) differences between the very young, young and teen children, and adults [146]. Secondly, a combination of macro- , meso- and micro-activities are often needed to fully address children exposures and are dependent on route of exposure, fate and transport of the contaminant of interest, and the timeframe of interest [9,19,147]. Dermal exposures, for example, require details (i.e., micro-activities) in contact activities such as frequency and duration of contact with surfaces and objects. The collection of such dermal activities can be labor intensive where videotaping and video-translation methodologies have been used to collect these micro-activities for children [147,148]. Thirdly, activity patterns can be influenced by a number of other factors such as the products in the environment and the affluence of the surroundings [15,149]. Therefore, country and environment differences offer us caution in using the activity patterns determined from one study to the other. Author Kwong et al. found that that the less affluent environment of Bangladesh children resulted in more contact with dirt floors than American children, for example, given the differences in the flooring of homes [149]. The EPA’s Child-Specific Exposure Factors Handbook and EPA’s Child-Specific Exposure Scenarios Examples offer general children’s activity patterns and exposure factors from a number of published studies, along with algorithmic approaches to address ingestion, inhalation, and dermal exposures and dose estimates in some contexts [150,151]. These documents were superseded by early versions of the *Exposure Factors Handbook* for all age groups [152].The researcher must appropriately relate these factors and algorithms to the exposure scenario, culture and climate, and measure or model relevant environmental concentrations of interest. Other countries offer activity pattern documents resulting from their local studies. Australia, for example, published the “Child Activity Patterns for Environmental Exposure Assessment in the Home” in 1999 based on numerous children of varying ages living in Port Pirie, South Australia. The work was completed through their Department of Human Services and used a combination of activity time diaries, video recorded behavior and questionnaires to look at activity patterns in homes. 

#### 3.3.2. Determining Environmental Concentrations

Accurate concentration estimates are needed for chemical and biological contaminants to accurately determine health risk for children. These concentrations can be measured or modeled. Proxy measurements, along with estimation techniques (e.g., dispersion model) offer means to determine environmental concentrations [15], where spatiotemporal resolution between environmental data and health data are important considerations to decrease uncertainty [153]. The closer researchers can get to measuring concentrations within proximate locations of where children spend time, the more accurate exposure estimates become; however there are time- and resource-intensive limitations [15]. 

#### 3.3.3. Biomarker Approach to Look at Exposure

Biomarkers (e.g., urine or blood analysis to look the compound or its metabolite concentration) are also used as an approach to look at environmental exposure, and often can offer a means of validation for especially aggregate (i.e., all routes) exposures where applicable [154]. Biomarkers of effect, however look at the response in the body (e.g., oxidative stress) to a compound or its metabolite offering a more direct look at an exposure-response outcome. Choosing the right biomarker of exposure/effect is critical when looking at environmental exposures for children and others. For example, Calafat et al. discussed the better use of urine as a biomarker for non-persistent, semi-volatile, hydrophilic chemicals such a phthalates and phenols [155]. In addition, a look at environmental media concentrations is often still required to identify sources of contaminants, to explore pathways of exposure, and to develop mitigation strategies. There are additional ethical considerations for children in biomarker studies related to collection and storage of samples, consent process, privacy, and proper communication of risk for a vulnerable population [156,157]. Nevertheless the field of environmental biomarker research has greatly expanded in the last 10–20 years to look at various contaminants of health outcomes. Of particular interest to this article is the expansion of the field where biomarkers are being used to look at early life stage exposures (i.e., before conception, during pregnancy, after birth) and to better understand the adverse mechanistic molecular changes following exposure to contaminants [158,159]. 

### 3.4. Risk Characterization and Risk Management

Risk characterization entails using the knowledge gained during hazard identification, dose-response assessment, and exposure assessment to make a determination of known risk and risk levels, in addition to addressing areas for further research, and ultimately making the information available to inform actionable policy recommendation for reducing or eliminating these hazards. In this final step, judgment is made about the nature and state of risks, given the uncertainties in the human risk assessment process. The EPA has prepared a risk characterization handbook adoptable for addressing risk characterization for children [160], and a framework document for assessing health risks of environmental exposures to children [14]. Risk characterization has varying considerations for characterizing risk for non-carcinogens and for carcinogens. Risk of exposure for non-carcinogens uses the dose estimate divided by the reference dose (RfD) (determined from dose-response curves) to determine a hazard index, while risk to carcinogens considers lifetime average daily doses (LADD) and potency factors to express population risk [118,161]. Risk from microbiological contaminants are defined through their corresponding dose-response curves which typically relate the probability of infection to the number of microbes to which a child is exposed. Usually the probability of infection is related to the number of microbes through an exponential function [162]. For children the characterization of risk to any contaminant must be done within the context of life-stages, and have a complete discussion surrounding any variability and uncertainty assessments conducted, and the reproducibility and biological plausibility factors considered in making policy recommendation [14]. Safety margins are set for children that consider uncertainty in measurement and extrapolations from animal studies, for example, and variability across the population [118]. Although we are interested in everyone’s chance of exposure and resulting outcome, particular attention is paid to those at greatest risk (i.e., upper end of a range of exposure and dose outcomes) [118]. 

**Figure 2:**
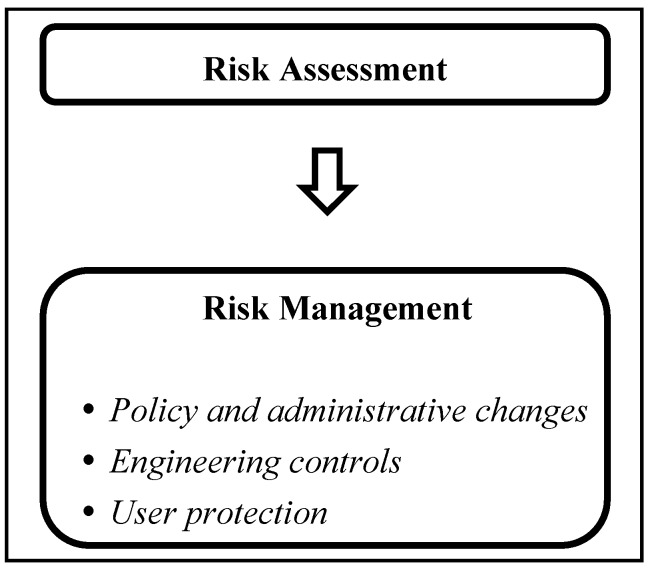
Risk management strategies once risks are assessed.

Risk management is a natural subsequent step following risk characterization, and uses the information from risk characterization in planning a mitigation strategy to reduce the exposure and ultimately improve health. As mentioned earlier, children are at greater risk either because of higher susceptibility or because they are subject to greater exposures. For mitigation, the focus is on reducing exposures. Mitigation strategies vary in their approach and can involve reducing the exposure by recommending administrative changes (e.g., chemical registration changes, chemical availability), engineering controls (e.g., product formulation, environmental release or cleanup and other issue dealing with fate and transport in the environment) and user protection changes (i.e., recommendation for behavioral change and communication to the public) (Figure 2). 

Mitigation strategies often involve policy change at the local, state or federal level. Implementation of change by an agency or organization is many times hindered by complexities and organizational barriers. A qualitative analysis on policy implementation was conducted in Mexico, and identified political resistance, communication difficulties, poor organization of power, and lack of strong regulatory platform as barriers to protecting children’s health, as opposed to scientific weight of evidence [163]. Those involved in children’s research globally may find these factors and others as impediments to varying degrees in implementing policies to protect children’s health. Improving children’s health globally will therefore require great effort among health, social, and political players, beyond the science of exposure and health.

In 1998, Goldman wrote about the importance of linking research and policy to ensure children’s environmental health, and much of that advice stands true today [164]. That publication stressed using the best science to inform policy, updating policy based on new research, making sure standards are strong enough to protect children, and expanding right-to-know education laws concerning children’s health, and in essence having the right leadership focused on prioritizing children’s health [164]. Although, great strides have been made to protect children’s health (e.g., setting air pollution standards and lead poisoning legislation in the United States), one of the greatest challenges, as Goldman saw it in 1998, was the implementation of more stringent policy for new compounds being released that could affect children’s health. In the United States, the Environmental Protection Agency maintains the Toxic Substance Control Act Inventory for existing chemicals manufactured, processed or imported. Today that list contains over 85,000 chemicals, a substantial growth from 62,000 in 1982 [165]. Many other chemicals that meet certain exemptions (e.g., low volume, low release, low exposure exemptions) do not make this inventory. Therefore, changing exposures and exposure to multiple chemicals, even at low levels, is a challenge that remains a concern for children’s health in 2017 and beyond. 

The European Union in 2007 passed comprehensive legislation for the registration, evaluation, authorization and restriction of chemicals (REACH) [166]. The legislation calls for the burden of proof of safety to lie with companies that produce chemicals used for industrial, manufacturing and daily uses (e.g., cleaning products), and hopes to ban products whose risks are unmanageable. This legislative action is seen as progressive and at the time there was hope that the United States will follow suit. In 2009, the U.S. EPA did introduce a strategic plan for evaluating the toxicity of chemicals, using a new screening initiative focused on in-vitro assays utilizing human cell lines, and looking at toxicity pathways-based approaches [167]. The U.S. EPA has also recently developed a new framework for the next generation of risk science (NexGen Framework) research with three core areas given the number of chemicals to be tested and the need for robust and timely assessments: recognition of multiple determinants that influence health (i.e., nutrition, socio-economic status), availability of data based on new toxicity pathways, and new risk assessment methodologies to improve risk management decisions [168]. Of particular interest in the NexGen Framework is the call for the expanded use of high throughput bio-monitoring data for exposure assessment, and the use of human cell lines to test for variability in the population. For children’s exposure to contaminants, we need to ensure these cell lines, bio-monitoring assessments, and all aspects of the nexGen Framework are representative of children’s exposures and outcomes. Recently, the United States, in a more aggressive attempt to evaluate the toxicity of chemicals allowed on the market and protect the public, introduced the Frank R. Lautenberg Chemical Safety Act of 2016 to amend the Toxic Substances Control Act (TSCA) with a greater focus on susceptible and highly exposed populations (e.g., pregnant women and children) [169]. The act calls for extended prioritized evaluation of some existing harmful chemicals by EPA, changes in risk based safety standards (i.e., affirmative finding on the safety of a new chemical must be established), increased public transparency and designated funding to implement this new approach, and is in keeping with the European REACH legislation approach. 

## 4. Conclusions

We have approached this review of children’s exposure to contaminants through the lens of the human risk assessment approach, and presented information from the literature on hazard assessment, dose-response, exposure assessment and risk characterization, with first a look at the exposure environment We also included additional thoughts on risk mitigation following risk characterization to address barriers to protecting children health beyond developing the scientific evidence. Research on children’s exposure to environmental contaminants often focuses in one area of the human risk assessment approach for a particular disease outcome or for a particular contaminant of interest. A variety of study designs are used, therefore, to address children’s exposures and health risks, and include prospective or retrospective epidemiological studies, and bio-monitoring or environmental and activity measurement studies, to name a few approaches. Each study has its benefits and limitations that most authors have articulated in order to better understand study results and implications for the field. The review also addressed some challenging and growing diseases of concern (e.g., autism, asthma) that likely have multiple determinants, offering opportunities for interdisciplinary research.

The burden of disease varies globally, due to differences in environmental, behavioral, and metabolic influences. In developed countries, such as the United States, average body mass index (BMI) among children (i.e., obesity) continues to grow, resulting in harmful effects such as high blood pressure and cholesterol, impaired emotional functioning, joint problems, and breathing problems [95]. Obesity, shown to produce changes in drug pharmacokinetics [103], could in turn affect a child’s already sensitive response to chronic and low concentrations of environmental contaminants. Children in developing countries continue to suffer greatly from malnutrition, air pollution, unsafe water, and poor sanitation (i.e., lack of hand washing facilities), resulting in multiple childhood diseases and adverse conditions. As such, research in children’s health and environmental exposures from varying regions will often be focused on their greater burdens of disease. Some developing countries are beginning to see changes in the environment and therefore changes in the environmental hazards that affect children. For example, Latin American and Caribbean governments have always been concerned with chemical and biological hazards from contaminated drinking water and indoor air hazards. Growing industrialization and urbanization over the last 50 years however have produced widespread and uncontrolled pollution, resulting in increased exposures to heavy metals, pesticides and electronic waste [4]. Great socio-economic disparities and the varying degree of modernization across Latin America further drives differences in health outcomes [4]. Within any developed or developing country, research shows that socio-economic disparities influence environmental exposures, and ultimately health outcomes, where minority and poorer populations tend to suffer the greater burden of environmental diseases [170].

Recently, a novel modeling framework has been developed to characterize and rank exposures in the United States based on research conducted under the National Children’s Study [120]. In essence, a number of databases are merged and computationally mined to look at aggregate and cumulative exposures to multiple chemical agents that might affect various health outcomes. Readers are encouraged to explore this tiered framework that incorporates national, state, and local information on the life cycle of chemicals, life stage analysis of individuals and populations (i.e., behavior biology), and information on biological effects of chemicals in order to develop the risk-relevance ranking of exposures [120]. This can be considered an overall risk assessment strategy for children, which although focused on chemicals, can be expanded to address biological contaminants. 

The field of children’s exposure to environmental contaminants continues to expand using sometimes older proven methodologies to address exposure and health outcome, and also newer methodologies given new techniques for field, lab and computer analysis. Of interest will be exposures to multiple chemicals at low dose and exposure to newer compounds that continue to enter the markets and therefore our environment. Researchers and policy makers must stay diligent and continue to look at innovative approaches to preemptively protect children, as they continue to discover causal factors for existing diseases.
